# More than Pneumonia: Distinctive Features of SARS-Cov-2 Infection. From Autopsy Findings to Clinical Implications: A Systematic Review

**DOI:** 10.3390/microorganisms8111642

**Published:** 2020-10-23

**Authors:** Stefano D’Errico, Martina Zanon, Martina Montanaro, Davide Radaelli, Francesco Sessa, Giulio Di Mizio, Angelo Montana, Salvatore Corrao, Monica Salerno, Cristoforo Pomara

**Affiliations:** 1 Department of Medical, Surgical and Health, University of Trieste, 34121 Trieste, Italy; martina.zanon@virgilio.it (M.Z.); martina.montanaro@virgilio.it (M.M.); davide_radaelli@hotmail.it (D.R.); 2 Department of Clinical and Experimental Medicine, University of Foggia, 71122 Foggia, Italy; francesco.sessa@unifg.it; 3 Department of Law, Forensic Medicine, Magna Graecia University of Catanzaro, 88100 Catanzaro, Italy; giulio.dimizio@unicz.it; 4 Department of Medical, Surgical and Advanced Technologies “G.F. Ingrassia”, University of Catania, 95121 Catania, Italy; angelomontana49@gmail.com (A.M.); monica.salerno@unict.it (M.S.); 5 Department of Internal Medicine, National Relevance and High Specialization Hospital Trust ARNAS Civico, Di Cristina and Benfratelli, 90100 Palermo, Italy; salvatore.corrao@unipa.it; 6 PROMISE Department, University of Palermo, 90100 Palermo, Italy

**Keywords:** COVID-19, autopsy, diffuse alveolar damage (DAD), acute respiratory distress syndrome (ARDS), microthrombosis, acute kidney injury (AKI), cytokine storm

## Abstract

Despite safety recommendations for the management of corpses with COVID-19 infection and the high number of deaths worldwide, the post-mortem investigation rate is extremely low as well as the scientific contributions describing the pathological features. The first results of post-mortem investigations provided interesting findings and contributed to promoting unexplored therapeutic approaches and new frontiers of research. A systematic review is provided with the aim of summarizing all autopsy studies up to February 2020 in which a complete post-mortem investigation in patients with COVID-19 disease was performed, focusing on histopathological features. We included case reports, case series, retrospective and prospective studies, letters to the editor, and reviews. A total of 28 studies fulfilled the inclusion criteria, producing a pooled dataset of 407 full autopsies. Analyzing the medical history data, only 12 subjects had died without any comorbidities (for 15 cases the data were not available). The post-mortem investigation highlighted that acute respiratory distress syndrome (ARDS) and multiple organ failure represent the main clinical features of COVID-19 disease, often leading to pulmonary thromboembolism and superimposed bronchopneumonia. The discussed data showed a strict relationship among the inflammatory processes, diffuse alveolar, and endothelial damage. In light of these results, the full autopsy can be considered as the gold standard to investigate unknown infections or pathogens resulting in death.

## 1. Introduction

Despite the fact that the COVID-19 infection is still ongoing in different countries worldwide, authorities and institutions are still limiting or avoiding autopsies of subjects who died from/with COVID-19 because of the possible biological hazards to personnel [1]. To improve safety in the management of cadavers confirmed or suspected of COVID-19 disease, scientific societies have provided guidelines and recommendations including safety measures for the safe and effective performance of HGC3 autopsy investigations (personnel protective equipment, negative-pressure autopsy rooms and limitations for operators) [2,3,4,5,6,7,8,9]. At the same time, several countries have changed their early recommendations, acknowledging the value of a complete post-mortem investigation in the control of the pandemic [10] Nevertheless, post-mortem investigation rates remain extremely low worldwide as well as the scientific contributions in the literature, indeed, the scientific community has called this the “lockdown of science” to define the reluctance toward autopsies [10,11,12,13,14]. It would have been sufficient to call for better and safer autopsies to clarify the full extent of organ involvement associated with COVID-19 [15] The absence of post-mortem investigations has failed to provide a valuable contribution to the correct management and treatment of patients, particularly in the first phase of the unknown infection. On the other hand, the execution of clinical and forensic autopsies has disclosed several important aspects of the disease, clarifying morphological and virologic features and promoting unexplored therapeutic approaches and new frontiers of research [16]. However, the limited number of autopsies did not establish the correct mortality rate of COVID-19 and did not define the statistical relevance of pathological findings, therefore, the risk of overestimation of pathologic features cannot be excluded.

## 2. Materials and Methods

### 2.1. Eligibility Criteria

The present systematic review was carried out according to the meta-analysis of observational studies in epidemiology (MOOSE) reporting standards [17]. We included all case reports, case series, retrospective and prospective studies, letters to the editors, and reviews that focused on COVID-19 and autopsy. The search was limited to human studies.

### 2.2. Search Criteria and Critical Appraisal

A systematic literature search and a critical appraisal of the collected studies were conducted. An electronic search of PubMed, ScienceDirect Scopus, and Excerpta Medica Database (EMBASE) from the inception of these databases to 17 August 2020 was performed. Search terms were “autopsy” OR “post-mortem” AND “Covid-19” OR “nCov-19” OR “SARS-CoV-2” in the title, abstract, and keywords. Cases in which a full autopsy was not performed (biopsies, mini-invasive approach) or in which histopathological investigations were not obtained were excluded because they did not meet the inclusion criteria. The bibliographies of all selected papers were examined and cross-referenced for further relevant literature. A methodological appraisal of each study was conducted according to the PRISMA standards including evaluation of bias. Data collection entailed study selection and data extraction. Three researchers (M.Z., M.M., D.R.) independently examined those papers whose title or abstract appeared to be relevant and selected the ones that analyzed the complete autopsy in people with the COVID-19 disease. Disagreements concerning eligibility between the three researchers were resolved by a consensus process. No unpublished or grey literature was searched. Data extraction was performed by one investigator (S.D.) and verified by two different investigators (C.P., M.S). This study was exempt from institutional review board approval as it did not involve human subjects.

## 3. Results

### 3.1. Search Results and Included Studies

An appraisal based on titles and abstracts as well as a manual search of reference lists was carried out. The reference lists of all selected articles were reviewed to detect still unidentified literature. A total of 32 studies fulfilled the inclusion criteria, producing a pooled dataset of 407 full autopsies. The reviewed studies involved a sample size ranging from one (i.e., case reports) to 80 autopsies (i.e., autopsy series). A multidisciplinary integrative post-mortem study within a sizeable group was not identifiable except for the study of Carsana (38 cases) [18], Bryce (67 cases) et al. [19], and Edler et al. (80 cases) [10]. Seven papers in which post-mortem investigations were limited to biopsies or mini-invasive approaches were excluded [20,21,22,23,24,25,26]. Autopsy series in which craniotomy and dissection of the central nervous system were avoided to minimize the risk of aerosol formation were excluded [27,28,29]. Contributions limited to ocular/retinal pathologic findings were excluded [30,31]. If the histopathologic examination was not performed, inclusion criteria were considered unsatisfied [32].

### 3.2. Study Characteristics

The following data were extracted from the included studies, summarizing in Table 1 the study source, country, full autopsy number, molecular analysis, and comorbidities.

The data referred to the post-mortem investigation such as the description of individual and structural safety measures, histological examination, immunohistochemistry, electron microscopy, and other investigations; focus on a single organ or apparatus or multiorgan collection of findings is summarized in Table 2.

### 3.3. Risk of Bias

This systematic review has a number of strengths that include the number and breadth of studies that span the globe, the manual search and scan of reference lists for the identification of all relevant studies, and a flowchart that describes in detail the study selection process. It must be noted that this review included studies that were published in a time frame of six months, thus, despite our efforts to fairly evaluate the existing literature, study results could be lacking in pre-printed reports. Additionally, the limited number of full autopsies and published papers may represent a limitation to the statistical value of the morphological findings and overestimating the significance of pathological features may become a risk. On the other hand, a case series needs to be considered as strong evidence and a standard to which a single experience may be reasonably compared.

### 3.4. Full Autopsy and Safety Protocol

This study identified 32 papers reporting experiences with 407 full autopsies in COVID-19-related death. In 16/32 papers, safety protocols (surgical scrub suit, surgical hat, eye protection glasses, FFP3 mask, waterproof gown including forearms, rubber boots, and cut-resistant gloves) and designed autopsy room for hazard group 3 pathogens (rapidly renewable positive-pressure ventilation and down-draft tables) were clearly stated [10,18,19,20,24,34,35,36,39,42,43,48,51,57,58,59].

### 3.5. Comorbidities

**Figure 1:**
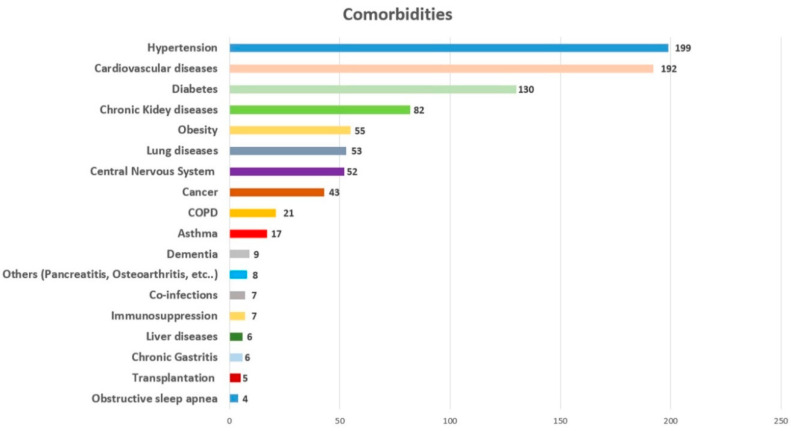
Summary of comorbidities.

Through this literature review, we analyzed 407 deaths from/with COVID-19 infection. The clinical history was available for 392 cases. It is important to note that only 12 subjects had died without known comorbidities, while in 380 cases, different comorbidities were reported, as summarized in Figure 1.

As reported in Figure 1, the most diffused comorbidities were hypertension, followed by cardiovascular diseases. Other important comorbidities were diabetes, chronic kidney disease, obesity, lung diseases, central nervous system diseases, and cancer. It is important to note that metabolic disorders and cardiovascular diseases were commonly found in the subjects who had died from/with COVID-19 presented in the reviewed papers.

### 3.6. Post-Mortem Investigations

The panel of post-mortem investigations varied among authors according to availability and resources. Hematoxylin and eosin stain was the routine approach for the histological study of organ samples and performed by all the authors. In 11/32 papers, hematoxylin and eosin stain was integrated with additional stains [18,24,34,36,39,40,45,49,50,53,57]. Histological analyses were completed with immunohistochemistry in 19/32 cases [11,18,19,24,33,34,35,38,47,48,49,52,53,55,56,57]. SARS-CoV-2 nucleocapsid protein monoclonal antibodies were used only in six papers [39,44,47,49,52,56]. SARS-CoV-2 spike protein antibodies were not included in the reviewed studies. The main immunohistochemical investigations concerned the lymphocellular population, macrophages, platelets, and megakaryocytes. Despite the involvement of cytokines and chemokines in the pathogenesis of COVID-19 disease, a panel for cytokines was proposed only in two studies [49,56]. A total of 12/32 studies completed the post-mortem investigation with electron microscopy [11,18,19,20,24,33,37,38,46,47,52,55]. The approach to post-mortem virologic analysis by real-time reverse transcription PCR resulted heterogeneous. Oropharyngeal and rhinopharyngeal swab were performed in all autopsy series. Tracheobronchial swabs were less represented. Rectal swabs were never performed.

### 3.7. Lungs

Heavy, congested, and edematous lungs were reported in all autopsy series. Pleural fluid was reported with high variability of volume as well as pleural adhesions [20,49,56]. Focal pulmonary areas of consolidation were frequently reported [19,20,24,34,35,56,58]. Massive pulmonary embolism was reported in some cases and represented the main cause of death [57]. Evidence of segmental or sub-segmental pulmonary emboli determining lobar infarction was also detected [11,19,20,39]. Vascular changes associated with proliferative and exudative diffuse alveolar damage with hyaline membrane deposition, necrosis of alveolar lining cells, TTF-1 positive type II pneumocytes hyperplasia with nucleomegaly, and prominent nucleoli alternatively combined with the accumulation of lymphocytes, macrophages, multinucleated giant cells were considered as distinctive features of COVID-19 infection [10,11,18,19,20,23,24,26,28,33,34,35,36,38,39,41,42,43,44,47,49,51,53,55,56,57,58]. Interstitial, peribronchiolar, and perivascular CD-3 positive T cells with a predominance of CD4 positive T cells over CD8-positive T cells were also described. CD20-positive B lymphocytes were generally absent, suggesting that the immune response in lung parenchyma is mainly mediated by T lymphocytes [18,20,33,34,35,38,49]. In the study of Wang et al., lymphocytic infiltration accounted for the majority of CD20 positive B cells, whereas CD3 positive T cells made up a small proportion including CD4 positive T cells and CD8 positive T cells [56]. Fibrin thrombi associated with increased CD61 positive platelets and megakaryocytes in pre-capillary and post-capillary vessels without complete luminal obstruction were also observed in lung specimens collected from patients with COVID-19 [18,19,33,38,43,47]. Thrombi were generally hetero-synchronous, showing different stages of organization [36]. In other cases, thrombotic features in large and small vessels were excluded [58]. Neutrophil infiltration in pulmonary vessels and extravasation of neutrophils into the alveolar space were described [18,38,59]. A higher expression of angiotensin-converting enzyme 2 (ACE2) in alveolar epithelial cells and capillary endothelial cells than in non-infected controls was observed in lung specimens [33]. Furthermore, perivascular and intra-alveolar ACE2-positive lymphocytes were recorded. A strong expression of chemokine and inflammatory cytokines (IL6, IL10, TNF-alpha) was observed when performed [56]. Prominent expression of the anti-viral antibodies on alveolar epithelial cells and on blood vessels was detected [56,60,61]. The contribution of electron microscopy was relevant to show architectural distortion in lungs injured by COVID-19 with new blood-vessel formation by intussusceptive angiogenesis and ultrastructural damage of the endothelium [19,33]. Round viral particles were found in type I and type II pneumocytes, alveolar macrophages, endothelial cells and in tracheal epithelial cells [18,19,44]. Viral aggregates were observed in the form of autophagosomes in the cytoplasm of type II pneumocytes [20].

### 3.8. Heart

Pathologic cardiac features were generally related to chronic cardiovascular comorbidities (dilated cardiomyopathy, atherosclerotic coronary artery disease, myocardial scarring). A few interstitial mononuclear inflammatory infiltrates that had a predominance of CD4+ T lymphocytes over CD8+ lymphocytes associated with acute myocyte necrosis were described in single cases [10,19,20,33,47,51]. Fulminant myocarditis was excluded in the widest case series of Lindner et al. [62]. Abnormalities of sinoatrial and atrioventricular conduction systems were excluded on random sections [11]. Intramyocardial venous thrombosis was observed in a few cases [47]. In situ hybridization of SARS-CoV-2 RNA confirmed the most likely localization of SARS-CoV-2 in the interstitial cells within the cardiac tissue or macrophages invading myocardial tissue. Signs of viral replication within myocardial tissue were also observed as well as an increased expression of cytokines, modulating the inflammatory response to viral infection in patients with a virus load above 1000 copies [62].

### 3.9. Kidneys

As previously described, evidence of acute tubular injury was described in COVID-19 patients; particularly, several modifications were detected in the proximal tubular epithelial cells such as variation of vacuolization size, loss of brush border, dilatation of the tubular lumen with cellular debris, and detachment of epithelium. All these modifications were related to viral toxicity [24,50,52]. In many cases, autolytic phenomena precluded the evaluation of acute kidney injury [20,43]. Arterionephrosclerosis, pre-existing pathological features of hypertension and diabetes, congestion of glomerular and peritubular capillaries and venules completed the renal findings [19,63]. Small fibrin platelet-rich thrombi in glomerular capillaries in association with severe injury of the endothelium were reported in a few cases as well as focal and sparse chronic inflammatory infiltrates [24,52]. Round viral particles were observed by electron microscopy in the tubular epithelium of the proximal tubules, rarely in the podocytes, and both individually or in aggregates in the cytoplasm or inside vesicles.

### 3.10. Central Nervous System (CNS)

Post-mortem abnormalities of the central nervous system were limited to a few single reports and included brain swelling and focal hemorrhagic lesions disseminated throughout cerebral hemispheric white matter with swollen axons at the periphery of the hemorrhagic foci, reactive gliosis, and oligondendrocyte apoptosis surrounding the lesions [48]. Cerebral pathologic features also included microthrombi and acute infarction, a focal parenchymal infiltrate of CD3 positive T-lymphocytes [19]. Viral particles in frontal lobe sections were detected by electron microscopy individually or in small vesicles of endothelial cells. Neural cell bodies also exhibited cytoplasmic vacuoles containing enveloped viral particles [46].

### 3.11. Other Organs

Gross autopsy findings also included hepato-splenomegaly and white pulp depletion as for SARS-CoV-2 [11,20,57]. In some cases, multifocal ischemic necrosis both in the periportal area (zone 1) and adjacent to terminal hepatic veins (zone 3) was described, showing neutrophilic infiltrates similar to ischemia-reperfusion injury as well as early organizing thrombi of the portal venules [19,43]. Numerous platelet-fibrin microthrombi were identified in hepatic sinusoids. Hepatic portal vein thrombosis was anecdotic [60,61,63,64,65]. Other organs were generally inconspicuous without significant histopathological features.

## 4. Discussion

Since the outbreak of COVID-19 disease, more than 355,000 people have died and only few papers have reported the pathological features from full autopsies on cadavers with this disease. The first reports dated to February 2020 came from China and were limited to gross examination [32]. In seven papers, post-mortem investigations were limited to biopsies or mini-invasive approaches [20,21,22,23,24,25,26]. In other studies, craniotomies and dissection of the central nervous system were avoided to minimize the risk of aerosol formation [27,28,29]. A complete “virtual” approach (virtopsy), an alternative to full autopsy, was also proposed as a possible safety measure [63]. At the end of this review, 32 papers described the pathological findings collected from 407 full autopsies and provided a worldwide methodological approach. Despite the diffidence toward post-mortem study of COVID-related deaths through autopsy and the limitations provided from authorities and institutions, European published experiences accounted for 55.52% of all full autopsies (226/407 autopsies) due to the early spread of the pandemic in this continent. The USA accounted for 37.83% of full reported autopsies (154/407) and only 7.12% (29/407) from China and Japan. Despite the high mortality rate, ranging from 1% to 10% in different countries according to extent of testing performed, histopathological data from autopsies are still limited [28,60,64].

As previously reported [66,67,68], the presence of comorbidities is an important factor to determine the clinical picture of COVID-19 patients. In this paper, the most common comorbidities were hypertension, cardiovascular diseases (such as coronary artery disease), diabetes, chronic kidney diseases, obesity, lung diseases, and cancer. These data are in accord with previous studies. Different reports have described an important relationship between COVID-19 severity outcome and hypertension [67] and cardiovascular diseases [68]. Recently, Rubino et al. [69] reported that diabetes is associated with an increased risk of severe COVID-19: the possible explanation is that this virus binds to angiotensin-converting enzyme 2 (ACE2) receptors, which are expressed in key metabolic organs and tissues such as pancreatic beta cells. Moreover, the ACE2 receptor is highly expressed in the kidneys: indeed, Henry and Lippi [70], based on their meta-analysis, reported a relation between chronic kidney diseases with enhanced risk of severe COVID-19 infection. In light of these data, it seems clear that metabolic disorders, cardiovascular diseases, and other severe comorbidities (for example cancer, lung and kidney diseases) can be considered as important clinical substrates in the severe outcome of COVID-19 patients.

Acute respiratory distress syndrome (ARDS) and multiple organ failure represent the main clinical features of COVID-19 disease, often leading to fatalities following pulmonary thromboembolism and superimposed bronchopneumonia [36,57]. All autopsy studies contributed to describe a similar pattern of diffuse alveolar damage (DAD) and severe endothelial damage, observing the effects of a pro-coagulative and pro-inflammatory state as the main pathologic features generally involved with a severe outcome. The observed morphologic pattern of diffuse alveolar damage and infiltrating perivascular lymphocytes are common with influenza A(H1N1) and SARS, while widespread vascular thrombosis with occlusion of alveolar capillaries, angiogenesis, and severe endothelial injury and intracellular SARS-CoV-2 represent distinctive features of SARS-CoV-2 [33]. It was supposed that early exudative/proliferative diffuse alveolar damage with interstitial lymphocytic infiltration and moderate to large presence of intra-alveolar macrophages could be associated with the sub-clinical phase, while symptoms that occurred in the organizing phase of damage in which features of fibrinous and organizing pneumonia with patchy distributed intra-alveolar fibrin balls were described [11,20,21,22,61]. Histopathological findings of pulmonary and systemic thrombotic events involving large to small vessels confirmed clinical evidence of the elevation of D-dimer and fibrin degradation products. The extensive nature of platelet-fibrin thrombi in small pulmonary arterial vessels might explain the severe hypoxemia observed in the early phase of the disease in ICU patients. Moreover, the high prevalence of pulmonary arterial thrombi described in the autopsy reports of patients with COVID-19 infection, and the absence of deep vein thrombosis suggested in situ formation [43,65,66]. Autopsy studies supported a causal relationship between the inflammatory processes involved with diffuse alveolar damage and endothelial damage.

A significant increase in angiotensin-converting enzyme 2 (ACE2) positive endothelial cells was observed in the lung as well as changes in endothelial morphology (disruption of intercellular junctions, cell swelling, loss of contact with the basal membrane). It is well known that ACE2, and putatively also sialic acids, represent the “doors” by which COVID-19 enters endothelial cells and pericytes; ACE2 receptors are ubiquitous, and are not only present in the endothelial cells of the alveolar membrane [71,72,73,74,75,76,77,78]. Virus-induced endothelitis is also important in vascular dysfunction and thrombotic events in the lung, kidney, and heart, and could be involved in the recent features of atypical Kawasaki disease observed in some children with COVID-19 [55]. Post-mortem investigations also supported the hypothesis of a direct entry of the virus into monocytes and macrophages through the ACE2 receptor on the surface of immune cells and demonstrated a crucial role of activated macrophages in the pathological development of the disease as well as the multi-organ failure observed in many patients, determining the release of cytokines (so called “cytokine storm”) [49,56,67]. It has been proposed that activated resident macrophages and pneumocytes initiate lung inflammatory response to SARS-CoV-2, overproducing proinflammatory cytokines and chemokines that are involved with endothelial cell apoptosis, vascular permeability, ARDS leading to pulmonary fibrosis, hypoxia, activation of complement, and the coagulation cascade leading to diffuse intravascular coagulation (DIC), myocardial dysfunction, and multiple organ failure [68]. The role of neutrophils also remained uncertain after autopsy studies. Neutrophilia has been associated with a poor prognosis in patients with COVID-19 and the neutrophil to lymphocyte ratio is an independent risk factor for worst outcomes [32,55,64]. As previously described, neutrophil extracellular traps (NETs) have the potential to propagate inflammation and microvascular thrombosis [78]. Future research should be addressed to provide evidence of SARS-CoV-2 to induce excessive NET formation by triggering a cascade of inflammatory reactions that promote microthrombosis and permanent damage to involved organs.

Analyzing the data concerning the heart, troponin elevation was observed in COVID-19 patients; this has been attributed in clinical studies to myocarditis, while only in a few cases to focal and epicardial lymphocyte infiltration. These findings were reported in cardiac samples so that other mechanisms of myocardial damage (ischemia, thrombosis of the microvasculature and cardiac veins, metabolic derangement) must be considered [10,11,19,20,38,47]. A role of infected pericytes causing capillary endothelial cells and microvascular dysfunction was hypothesized to explain individual cardiomyocyte necrosis. Clinical and post-mortem studies failed to demonstrate viral particles in cardiomyocytes and endothelial cells; moreover, the presence of viral particles in myocardial interstitial cells was interpreted as evidence of a viremic phase of the disease or of the migration of macrophages from the lungs [79].

Acute kidney injury (AKI) in patients with COVID-19 is associated with higher overall mortality compared with that of patients without renal involvement [80,81]. Renal dysfunction was described in COVID-19 patients evolving into severe renal disease and acute renal failure in 3–7% of cases [55]. It was observed that the severity of pneumonia could be an independent risk factor in the development of acute kidney injury and renal complications in patients with COVID-19 [81]. Post-mortem studies showed different degrees of acute tubular necrosis, luminal brush border sloughing, hyaline casts, microthrombi, and mild interstitial fibrosis, which are consistent with the high proportion of renal tubular protein in urine observed in clinical studies and suggest a direct virulence of SARS-CoV-2. Acute (proximal) tubular injury was described in autopsy series and viral particles and ultrastructural analysis revealed abundant round viral particles in the epithelial tubular cells and podocytes where ACE-2 receptors are mainly expressed [52,71]. A possible role of CD147 in SARS-CoV-2 infection of renal epithelial tubular cells has been proposed. CD147 is a transmembrane glycoprotein highly expressed on the cell surface of the proximal tubular epithelium involved with cyclophillins in the replication process of COVID-19 and represents a promising strategy for therapy. Ischemic and cytokine related acute kidney injury was alternatively supposed [37].

COVID-19 infection may also cause neurological diseases. Autopsy studies have investigated central nervous system damage. The post-mortem evidence showed the presence of viral particles in neuronal and endothelial cells of the frontal lobe sections in COVID-19 patients with neurologic and psychiatric symptoms. Hematogenous and neuronal retrograde routes have been proposed to explain the entry of neurotropic respiratory viruses into the CNS. The pathobiology of these neuro-invasive viruses is still not completely known, and it is therefore important to explore the impact of CoV infections on the nervous system [79,82,83,84,85].

Furthermore, liver damage was evaluated: the pathogenesis of the hepatic damage described in autopsy studies is controversial and could be related to a direct cytopathic effect of SARS-CoV-2, to the effect of drugs, or to underlying liver disease. A reaction to hyperinflammation as well as hypoxia could not be excluded [28,85]. The identification of hemophagocytic histiocytes in lymph nodes, spleen, and the liver is consistent with a hyper-inflammatory state and with virus-induced macrophage activation syndrome.

Finally, it is important to consider the presence of several important comorbidities in 96.93% (380/392) of all discussed cases. In light of this consideration, “which came first: the chicken or the egg?” In other words, are the post-mortem findings related to the COVID-19 infection? It would be better to clarify this in future studies, as the question is still not fully answered.

## 5. Conclusions

Autopsies contribute to a better understanding of the pathological mechanisms of the disease and support evaluation and management of surviving patients. The limited number of full autopsies and published papers may represent a limitation to the statistical value of the morphological findings and overestimating the significance of pathologic features may become a risk. On the other hand, the analysis of case series could reduce this bias.

As shown in this review, to further comprehend the pathological pathways of COVID-19, many autopsies should be performed including patients of different ages and with various comorbidities. Each country has chosen to respond to this emergency with its own decisions: on one hand, countries such as Italy have chosen to not perform hospital autopsies; on the other hand, countries such as Germany have ordered mandatory autopsies on all patients who die with a diagnosis of COVID-19. Particularly, in Germany, autopsies have been performed on COVID-19 patients even if the cause of death seemed clear [56]. In our opinion, as also remarked by Barth et al. [15], on the basis of the tragically increasing number of deaths of COVID-19 patients, autopsy should be considered mandatory, particularly in the case of unknown diseases.

Specimens collected during autopsy represent an invaluable source for potential further research to better define controversial aspects of SARS-CoV-2 toxicity, endocellular viral replication, and influence new potential therapeutic strategies also in those patients who have been infected by the virus with a hospitalization period in the intensive care unit who are still living (so called “COVID-19 survivors”) [13,39]. Actually, efforts are addressed toward investigating the genetic susceptibility to COVID-19 and prognostic value of specific loci involvement [86]. We firmly agree with the statement that in order to better contain the outbreak, a broader effort is required to help physicians fighting this pandemic, not only in improving the capacity of health systems to prevent death by the use of proper treatments, but by allowing physicians to learn from the dead. In conclusion, full autopsies must be encouraged and considered as a priority for the scientific community. Life after death. The myth goes on.

## Figures and Tables

**Table 1:** General data obtained by analyzing the studies included in this literature review.

Source	Country	Full Autopsy (Number and Sex)	Molecular Analysis	Comorbidities
Ackermann et al. [33]	Europe (Germany)	7 (2F; 5M)	NR	Hypertension (2F, 5M); Diabetes, type II (3M); Immunosuppression (1M)
Aguiar et al. [34]	Europe (Switzerland)	1 (F)	RT-PCR	Morbid obesity
Barton et al. [35]	USA (OK)	2 (M)	RT-PCR	Hypertension (1); Deep vein thrombosis (1); Remote pancreatitis (1); Osteoarthritis (1); Myotonic muscular dystrophy (1)
Bradley et al. [20]	USA (WA)	14 (8F; 6M)	RT-PCR	Type 2 diabetes (4F, 2M); Hypertension (5F, 5M); Obstructive sleep apnea (1F, 3M); Obesity(3F, 2M); Cardiovascular diseases (4F, 2M); Chronic kidney disease (4F, 3M); Traumatic brain injury (1M); Frontotemporal dementia (1M); Congestive heart failure (3F, 1M); Hypothyroidism (1F); Osteoporosis (2F); Deep vein thrombosis(1F); Breast cancer (2F)
Bryce et al. [19]	USA (NY)	67 (not available)	RT-PCR	Hypertension (42); Diabetes mellitus (27); Cardiovascular diseases (40); Chronic kidney disease (18); Asthma (12); Obesity (8); Co-infections (7); Cancer (5); Transplantation (5); Chronic obstructive pulmonary disease (COPD) (4).
Buja et al. [11]	USA (TX)	3 (3M)	RT-PCR	Obesity (2M); Hypertension (1M); Heart failure (1M); Type II diabetes mellitus (1M); Microcytic anemia (1M)
Carsana et al. [18]	Europe (Italy)	38 (5F; 33M)	RT-PCR	data were available for 31 patients: Diabetes (9); Hypertension (18); Past malignancies (4); Cardiovascular disorders (11); COPD (3)
Cipolloni et al. [36]	Europe (Italy)	2 (2M)	RT-PCR	Data were not available (1M); nil (1M)
Edler et al. [10]	Europe (Germany)	80 (34F; 46M)	RT-PCR	Cardiovascular diseases (68); Lung diseases (44); Kidney diseases (27); Central nervous system (CNS) diseases (28); Diabetes mellitus (17); Carcinomas/hematological diseases (13); 2 F: nil.
Farkash et al. [37]	USA (MI)	1 (M)	RT-PCR	Obesity; Hyperlipidemia
Fox et al. [38]	USA (LA)	10 (not available)	RT-PCR	Hypertension (7), Type II diabetes mellitus (5); chronic kidney disease (1); Thyroidectomy (1); Rheumatoid arthritis (1); Polymyositis (1); Atrial fibrillation (1); End-stage renal disease (1); Not available (1)
Grosse et al. [39]	Europe (Austria)	14 (5F; 9M)	RT-PCR	Heart disease (5F; 9M); Hypertension (3F; 5M); Renal disease (4F; 2M); Neurologic disease (4F; 3M); Diabetes mellitus (2F; 2M); Malignancy (2M); Respiratory disease (5M); Chronic gastritis (3F; 3M); Liver cirrhosis/fibrosis (1M).
Iuga et al. [40]	USA (NY)	5 (1F; 4M)	NR	Hypertension (5); Diabetes (3); Ischemic cardiomyopathy (3); Chronic lung disease (2); Chronic obstructive pulmonary disease (1); Interstitial lung disease (1); Prostate carcinoma (2); recent spinal surgery (1).
Konopka et al. [41]	USA (MI)	1	NR	Asthma; Diabetes mellitus
Lacy et al. [42]	USA (MI)	1	RT-PCR	Diabetes mellitus, Obesity, Hyperlipidemia, Mild intermittent asthma
Lax et al. [43]	Europe (Austria)	11 (not available)	RT-PCR	Obesity (2); Hypertension (9); Type 2 diabetes mellitus (5); Cerebrovascular disease (4); Dementia (4); COPD (1); Coronary heart disease(1); History of malignant disease (1); Pulmonary embolism (1)
Menter et al. [44]	Europe (Switzerland)	21 (4F; 17M)	RT-PCR	Hypertension (21); Cardiovascular disease (15); Obesity (19); Diabetes mellitus (7); Chronic neurological condition (5); COPD (3); Malignancy (3); Chronic liver disease (2); Chronic kidney disease (4); Acquired immunosuppression (1)
Okudela et al. [45]	Japan	1 (F)	RT-PCR	not available
Paniz Mondolfi et al. [46]	USA (NY)	1 (M)	RT-PCR	Parkinson’s disease
Reichard et al. [47]	USA (MN)	1 (M)	RT-PCR	Atherosclerosis
Remmelink et al. [48]	Europe (Belgium)	17 (5F; 12M)	RT-PCR	Hypertension (6F; 4M); Chronic renal failure (2F; 1M); Liver cirrhosis (2F; 1M); Coronary artery disease (2F; 2M); Cerebrovascular disease (2F; 2M); Diabetes (4F; 6M); COPD (2M); Cancer (1F; 3M); Nil (2)
Santoriello et al. [49]	USA (NY)	42 (13F; 29M)	NR	Hypertension (30); Diabetes mellitus (17); Coronary artery disease or Cerebrovascular accident (13); Immunosuppression (2); Chronic Kidney diseases (8); Obesity (10);
Schaller et al. [50]	Europe (Germany)	12 (5F; 7M)	RT-PCR	Immunosuppression (2); Hypertension (7); COPD (2); Chronic Kidney diseases (3); Obesity (2); Cardiovascular diseases (5); Adenocarcinoma of the lung (1); Dementia (1)
Su et al. [51]	China	26 (7F; 19M)	NR	Lung cancer (1M; 1F); Pancreas cancer (1M); Gastric cancer (1M); Liver cancer (1M); Skin Cancer (1M); Diabetes mellitus (2M; 1F); Hypertension (7M; 4F); Chronic kidney diseases (1M; 1F); Nil: 4 (2M; 2F); not available 6 (5M; 1F);
Suess et al. [52]	Europe (Germany)	1 (M)	RT-PCR	Hypertension; Diabetes mellitus;
Tombolini et al. [53]	Europe (Italy)	2 (F)	RT-PCR	Hashimoto’s thyroiditis (1); Diabetes mellitus (1); Nil (1)
Varga et al. [54]	Europe (Switzerland)	3 (1F; 2M)	NR	Coronary artery disease (1M); Hypertension (1F; 2M); Diabetes (1F); Obesity (1F).
Wang et al. [55]	China	2 (1F; 1M)	RT-PCR	Type 2 diabetes (F); Hypertension (F); Nil (M)
Wichmann et al. [56]	Europe (Germany)	12 (3F; 9M)	RT-PCR	Obesity (3M); Parkinson disease (2M); Chronic kidney diseases (1M; 1F); Hypertension (3M); Diabetes mellitus (3M); Asthma (2M); Dementia (1F); Epilepsy (1F); Trisomy 21 (1F); Cardiovascular diseases (6M; 3F); Lung cancer (1F); COPD (1F)
Youd et al. [57]	Europe (UK)	9 (5F; 4M)	RT-PCR	Diabetes (3F; 1M); Hypertension (2F; 2M); COPD (1F; 3M); Asthma (1F); Cardiovascular diseases (3M; 1F); Dementia (1F; 1M); Parkinson’s disease (1M); HIV (1M)

**Table 2:** Summary of post-mortem examination.

Source	Safety Measures	Histological Examination	Immunohistochemistry	Electron Microscopy	Others (Immunofluorescence, RNA-In Situ Hybridization, Post-Mortem Biochemistry)	Focus
[33]	NR	H&E	ACE2; CD3; CD4; CD8; CD15	Y	NR	Lungs
[34]	Y	H&E; SFOG	pankeratin; CD68; CD3	NR	Post-mortem biochemistry (PCR, procalcitonin)	Full
[35]	Y	H&E	CD3; CD4; CD8; CD20; CD68	NR	NR	Full
[20]	Y	H&E	NR	Y	NR	Full
[19]	Y	H&E	CD61; CD3; CD4; CD8; ACE2	Y	Immunofluorescence; RNA-ISH	Full
[11]	NR	H&E	CD3; CD4; CD8; CD68; TTF1; CK-7; p40; CK5/6	Y	NR	Full
[18]	Y	H&E; Masson	CD68; CD3; CD45; CD61; TTF1; p40; MIB-1	Y	NR	Full
[36]	Y	H&E; Masson	CD4, CD8, CD20, CD68, CD79 Factor VIII, TNF-alpha, IL6, ACE2 SARS nucleo-capsid protein	NR	NR	Lungs
[10]	Y	H&E	NR	NR	RNA-ISH	Full
[37]	NR	NR	NR	Y	NR	Kidneys
[38]	NR	H&E	CD4; CD8; CD61; CD31	Y	Immunofluorescence	Heart, lungs
[39]	Y	H&E, van Gieson, PAS, Ziehl Nielsen, Grocott methenamine	CD3, CD20, CD68, TTF-1, pancytokeratine	NR	NR	Lungs
[40]	NR	H&E; Masson	NR	NR	NR	Adrenal glands
[41]	NR	H&E	NR	NR	NR	Lungs
[42]	Y	NR	NR	NR	NR	Full
[43]	Y	H&E	NR	NR	NR	Full
[44]	Y	H&E; aniline blue; Giemsa; periodic acid-Schiff; Congo red; Prussian blue; rhodamine; Gram, Brown-Brenn; Grocott methenamine silver stain	CD3; CD4; CD8; CD20; CD68; MUM1; TTF1; fibrin; ATTR	Y	NR	Full
[45]	NR	H&E	NR	NR	NR	Full
[46]	NR	NR	NR	Y	NR	CNS
[47]	NR	H&E; LFB/PAS; PLP	GFAP; APP	NR	NR	CNS
[48]	Y	H&E, Masson trichrome, PAS, Gomori-Grocott	Anti-CMV, anti-HSV, anti-Pneumocystis, nucleo-capsid protein	NR	NR	Full
[49]	NR	H&E	NR	NR	ISH	Kidneys
[50]	Y	H&E	NR	NR	NR	Full
[51]	NR	H&E	CD61; CD31; ACE2; SARS nucleo-capsid protein	Y	Immunofluorescence	Kidneys
[52]	NR	H&E; PAS	TTF1: CD68	NR	NR	Full
[53]	NR	H&E	NR	NR	NR	Full
[54]	NR	H&E	caspase 3	Y	NR	Full
[55]	Y	H&E; AB-PAS	CD68; CD3; CD4; CD8; CD20; CD56; PD-1; PD-L1; IL6; IL10; TNF-alpha; ACE-2; SARS-CoV2 Rp3 N protein	NR	NR	Full
[56]	NR	H&E	cytokeratin AE1/AE3	NR	NR	Full
[57]	Y	H&E	NR	NR	NR	Full

NR: not reported; H&E: hematoxylin and eosin; SFOG: acid fuchsin-orange G stain; GFAP: glial fibrillary acidic protein; APP: amyloid precursor protein; LFB/PAS: Luxol fast blue/periodic acid-Schiff; PLP: myelin proteolipid protein; AB-PAS: Alcian blue-periodic acid-Schiff; PCR: protein C reactive; TTF1: thyroid transcription factor; ACE-2: angiotensin converting enzyme 2; TNF-alpha: tumor necrosis factor-alpha; CK: cytokeratin; MIB-1: monoclonal antibody to Ki67; MUM1: Multiple Myeloma-1; ATTR: antibodies against transthyretin.

## References

[1] Pomara C., Volti G.L., Cappello F. (2020). COVID-19 Deaths: Are We Sure It Is Pneumonia? Please, Autopsy, Autopsy, Autopsy!. J. Clin. Med..

[2] Centers for Disease Control and Prevention Collection and Submission of Postmortem Specimens from Deceased Persons with Known or Suspected COVID-19. https://eaaf.org/wp-content/uploads/covid19-PDFs/EEUU/CDC-guidance-postmortem-specimens.pdf.

[3] Osborn M., Lucas S.B., Stewart R., Swift B., Youd E. Autopsy Practice Relating to Possible Cases of COVID-19 (2019-nCov, Novel Coronavirus from China 2019/2020). https://www.rcpath.org/uploads/assets/d5e28baf-5789-4b0f-acecfe370eee6223/447e37d0-29dd-4994-a11fe27b93de0905/Briefing-on-COVID-19-autopsy-Feb-2020.pdf.

[4] Basso C., Calabrese F., Sbaraglia M., Del Vecchio C., Carretta G., Saieva A., Donato D., Flor L., Crisanti A., Tos A.P.D. (2020). Feasibility of postmortem examination in the era of COVID-19 pandemic: The experience of a Northeast Italy University Hospital. Virchows Arch..

[5] Fineschi V., Aprile A., Aquila I., Arcangeli M., Asmundo A., Bacci M., Cingolani M., Cipolloni L., D’Errico S., de Casamassimi I. (2020). Management of the corpse with suspect, probable or confirmed COVID-19 respiratory infection—Italian interim recommendations for personnel potentially exposed to material from corpses, including body fluids, in morgue structures, during autopsy practice. Pathol. J. Ital. Soc. Anat. Pathol. Diagn. Cytopathol..

[6] Santurro A., Scopetti M., D’Errico S., Fineschi V. (2020). A technical report from the Italian SARS-CoV-2 outbreak. Postmortem sampling and autopsy investigation in cases of suspected or probable COVID-19. Forensic Sci. Med. Pathol..

[7] Hanley B., Lucas S.B., Youd E., Swift B., Osborn M. (2020). Autopsy in suspected COVID-19 cases. J. Clin. Pathol..

[8] Sapino A., Facchetti F., Bonoldi E., Gianatti A., Barbareschi M. (2020). The autopsy debate during the COVID-19 emergency: The Italian experience. Virchows Arch..

[9] Keten D., Okdemir E., Keten A. (2020). Precautions in postmortem examinations in Covid-19 - Related deaths: Recommendations from Germany. J. Forensic Leg. Med..

[10] Edler C., Schröder A.S., Aepfelbacher M., Fitzek A., Heinemann A., Heinrich F., Klein A., Langenwalder F., Lütgehetmann M., Meißner K. (2020). Dying with SARS-CoV-2 infection—An autopsy study of the first consecutive 80 cases in Hamburg, Germany. Int. J. Legal Med..

[11] Buja L.M., Wolf D., Zhao B., Akkanti B., McDonald M., Lelenwa L., Reilly N., Ottaviani G., Elghetany M.T., Trujillo D.O. (2020). The emerging spectrum of cardiopulmonary pathology of the coronavirus disease 2019 (COVID-19): Report of 3 autopsies from Houston, Texas, and review of autopsy findings from other United States cities. Cardiovasc. Pathol..

[12] Salerno M., Sessa F., Piscopo A., Montana A., Torrisi M., Patanè F., Murabito P., Li Volti G., Pomara C. (2020). No Autopsies on COVID-19 Deaths: A Missed Opportunity and the Lockdown of Science. J. Clin. Med..

[13] Pomara C., Li Volti G., Cappello F. (2020). The post-lockdown era: What is next in Italy?. Front. Pharmacol..

[14] Tzankov A., Jonigk D. (2020). Unlocking the lockdown of science and demystifying COVID-19: How autopsies contribute to our understanding of a deadly pandemic. Virchows Arch..

[15] Barth R.F., Xu X., Buja L.M. (2020). A Call to Action: The Need for Autopsies to Determine the Full Extent of Organ Involvement Associated With COVID-19. Chest.

[16] Püschel K., Sperhake J.P. (2020). Corona deaths in Hamburg, Germany. Int. J. Legal Med..

[17] Stroup D.F., Berlin J.A., Morton S.C., Olkin I., Williamson G.D., Rennie D., Moher D., Becker B.J., Sipe T.A., Thacker S.B. (2000). Meta-analysis of observational studies in epidemiology: A proposal for reporting. J. Am. Med. Assoc..

[18] Carsana L., Sonzogni A., Nasr A., Rossi R.S., Pellegrinelli A., Zerbi P., Rech R., Colombo R., Antinori S., Corbellino M. (2020). Pulmonary post-mortem findings in a series of COVID-19 cases from northern Italy: A two-centre descriptive study. Lancet Infect. Dis..

[19] Bryce C., Grimes Z., Pujadas E., Ahuja S., Beasley M.B., Albrecht R., Hernandez T., Stock A., Zhao Z., Al Rasheed M. (2020). Pathophysiology of SARS-CoV-2: Targeting of endothelial cells renders a complex disease with thrombotic microangiopathy and aberrant immune response. The Mount Sinai COVID-19 autopsy experience. medRxiv.

[20] Bradley B.T., Maioli H., Johnston R., Chaudhry I., Fink S.L., Xu H., Najafian B., Deutsch G., Lacy J.M., Williams T. (2020). Histopathology and ultrastructural findings of fatal COVID-19 infections in Washington State: A case series. Lancet.

[21] Giacca M., Bussani R., Schneider E., Zentilin L., Collesi C., Ali H., Braga L., Secco I., Volpe M.C., Colliva A. (2020). Persistence of viral RNA, widespread thrombosis and abnormal cellular syncytia are hallmarks of COVID-19 lung pathology. medRxiv.

[22] Copin M.C., Parmentier E., Duburcq T., Poissy J., Mathieu D., The Lille COVID-19 ICU and Anatomopathology Group (2020). Time to consider histologic pattern of lung injury to treat critically ill patients with COVID-19 infection. Intensive Care Med..

[23] Duarte-Neto A.N., de Almeida Monteiro R.A., da Silva L.F.F., Malheiros D.M.A.C., de Oliveira E.P., Theodoro Filho J., Pinho J.R.R., Soares Gomes-Gouvêa M., Salles A.P.M., de Oliveira I.R.S. (2020). Pulmonary and systemic involvement of COVID-19 assessed by ultrasound-guided minimally invasive autopsy. Histopathology.

[24] Menter T., Haslbauer J.D., Nienhold R., Savic S., Hopfer H., Deigendesch N., Frank S., Turek D., Willi N., Pargger H. (2020). Post-mortem examination of COVID19 patients reveals diffuse alveolar damage with severe capillary congestion and variegated findings of lungs and other organs suggesting vascular dysfunction. Histopathology.

[25] Xu X., Chang X.N., Pan H.X., Su H., Huang B., Yang M., Luo D.J., Weng M.X., Ma L., Nie X. (2020). Pathological changes of the spleen in ten patients with coronavirus disease 2019(COVID-19) by postmortem needle autopsy. Zhonghua Bing Li Xue Za Zhi Chin. J. Pathol..

[26] Yan L., Mir M., Sanchez P., Beg M., Peters J., Enriquez O., Gilbert A. (2020). Autopsy Report with Clinical Pathological Correlation. Arch. Pathol. Lab. Med..

[27] Dell’Aquila M., Cattani P., Fantoni M., Marchetti S., Aquila I., Stigliano E., Carbone A., Oliva A., Arena V. (2020). Postmortem swabs in the Sars-CoV-2 Pandemic: Report on 12 complete clinical autopsy cases. Arch. Pathol. Lab. Med..

[28] Bösmüller H., Traxler S., Bitzer M., Häberle H., Raiser W., Nann D., Frauenfeld L., Vogelsberg A., Klingel K., Fend F. (2020). The evolution of pulmonary pathology in fatal COVID-19 disease: An autopsy study with clinical correlation. Virchows Arch..

[29] Conde P.N., Monraval P.A., Medina C.M., Sánchez A.J., Teruel J.C., Marco J.F., Santos V.P., Aranda E.M. (2020). Mayordomo Aranda, E. Autopsy findings from the first known death from Severe Acute Respiratory Syndrome SARS-CoV-2 in Spain. Rev. Esp. Patol..

[30] Casagrande M., Fitzek A., Püschel K., Aleshcheva G., Schultheiss H.P., Berneking L., Spitzer M.S., Schultheiss M. (2020). Detection of SARS-CoV-2 in Human Retinal Biopsies of Deceased COVID-19 Patients. Ocul. Immunol. Inflamm..

[31] Löffler K.U., Reinhold A., Herwig-Carl M.C., Tzankov A., Holz F.G., Scholl H.P.N., Meyer P. (2020). Ocular post-mortem findings in patients having died from COVID-19. Ophthalmologe.

[32] Liu Q., Wang R.S., Qu G.Q. (2020). Gross Examination Report of a COVID-19 Death Autopsy. J. Forensic Med..

[33] Ackermann M., Verleden S.E., Kuehnel M., Haverich A., Welte T., Laenger F., Vanstapel A., Werlein C., Stark H., Tzankov A. (2020). Pulmonary Vascular Endothelialitis, Thrombosis, and Angiogenesis in Covid-19. N. Engl. J. Med..

[34] Aguiar D., Lobrinus J.A., Schibler M., Fracasso T., Lardi C. (2020). Inside the lungs of COVID-19 disease. Int. J. Legal Med..

[35] Barton L.M., Duval E.J., Stroberg E., Ghosh S., Mukhopadhyay S. (2020). COVID-19 Autopsies, Oklahoma, USA. Am. J. Clin. Pathol..

[36] Cipolloni L., Sessa F., Bertozzi G., Baldari B., Cantatore S., Testi R., D’Errico S., Di Mizio G., Asmundo A., Castorina S. (2020). Preliminary Post-Mortem COVID-19 Evidence of Endothelial Injury and Factor VIII Hyperexpression. Diagnostics.

[37] Farkash E.A., Wilson A.M., Jentzen J.M. (2020). Ultrastructural Evidence for Direct Renal Infection with SARS-CoV-2. J. Am. Soc. Nephrol..

[38] Fox S.E., Akmatbekov A., Harbert J.L., Li G., Brown J.Q., Vander Heide R.S. (2020). Pulmonary and cardiac pathology in African American patients with COVID-19: An autopsy series from New Orleans. Lancet Respir. Med..

[39] Grosse C., Grosse A., Salzer H., Dünser M., Motz R., Langer R. (2020). Analysis of cardiopulmonary findings in COVID-19 fatalities: High incidence of pulmonary artery thrombi and acute suppurative bronchopneumonia. Cardiovasc. Pathol..

[40] Iuga A.C., Marboe C.C., Yilmaz M.M., Lefkowitch J.H., Gauran C., Lagana S.M. (2020). Adrenal Vascular Changes in COVID-19 Autopsies. Arch. Pathol. Lab. Med..

[41] Konopka K.E., Wilson A., Myers J.L. (2020). Postmortem Lung Findings in an Asthmatic Patient With Coronavirus Disease 2019. Ann Oncol.

[42] Lacy J.M., Brooks E.G., Akers J., Armstrong D., Decker L., Gonzalez A., Humphrey W., Mayer R., Miller M., Perez C. (2020). COVID-19: POSTMORTEM DIAGNOSTIC AND BIOSAFETY CONSIDERATIONS. Am. J. Forensic Med. Pathol..

[43] Lax S.F., Skok K., Zechner P., Kessler H.H., Kaufmann N., Koelblinger C., Vander K., Bargfrieder U., Trauner M. (2020). Pulmonary Arterial Thrombosis in COVID-19 With Fatal Outcome: Results From a Prospective, Single-Center, Clinicopathologic Case Series. Ann. Intern. Med..

[44] Okudela K. (2020). CASE REPORT A Japanese case of COVID-19: An autopsy report. Pathol. Int..

[45] Paniz-Mondolfi A., Bryce C., Grimes Z., Gordon R.E., Reidy J., Lednicky J., Sordillo E.M., Fowkes M. (2020). Central nervous system involvement by severe acute respiratory syndrome coronavirus-2 (SARS-CoV-2). J. Med. Virol..

[46] Reichard R.R., Kashani K.B., Boire N.A., Constantopoulos E., Guo Y., Lucchinetti C.F. (2020). Neuropathology of COVID-19: A spectrum of vascular and acute disseminated encephalomyelitis (ADEM)-like pathology. Acta Neuropathol..

[47] Remmelink M., De Mendoca R., D’Haene N., De Clercq S., Verocq C., Lebrun L., Lavis P., Racu M.L., Trepant A.L., Maris C. (2020). Unspecific post-mortem findings despite multiorgan 1 viral spread in COVID-19 patients. medRxiv.

[48] Santoriello D., Khairallah P., Bomback A.S., Xu K., Kudose S., Batal I., Barasch J., Radhakrishnan J., D’Agati V., Markowitz G. (2020). Postmortem Kidney Pathology Findings in Patients with COVID-19. J. Am. Soc. Nephrol..

[49] Schaller T., Hirschbühl K., Burkhardt K., Braun G., Trepel M., Märkl B., Claus R. (2020). Postmortem Examination of Patients with COVID-19. JAMA.

[50] Su H., Yang M., Wan C., Yi L.X., Tang F., Zhu H.Y., Yi F., Yang H.C., Fogo A.B., Nie X. (2020). Renal histopathological analysis of 26 postmortem findings of patients with COVID-19 in China. Kidney Int..

[51] Suess C., Hausmann R. (2020). Gross and histopathological pulmonary findings in a COVID-19 associated death during self-isolation. Int. J. Legal Med..

[52] Tombolini A., Scendoni R. (2020). SARS-CoV-2-related deaths in routine forensic autopsy practice: Histopathological patterns. Int. J. Legal Med..

[53] Varga Z., Flammer A.J., Steiger P., Haberecker M., Andermatt R., Zinkernagel A.S., Mehra M.R., Schuepbach R.A., Ruschitzka F., Moch H. (2020). Endothelial cell infection and endotheliitis in COVID-19. Lancet.

[54] Wang C., Xie J., Zhao L., Fei X., Zhang H., Tan Y., Nie X., Zhou L., Liu Z., Ren Y. (2020). Alveolar macrophage dysfunction and cytokine storm in the pathogenesis of two severe COVID-19 patients. EBioMedicine.

[55] Wichmann D., Sperhake J.-P., Lütgehetmann M., Steurer S., Edler C., Heinemann A., Heinrich F., Mushumba H., Kniep I., Schröder A.S. (2020). Autopsy Findings and Venous Thromboembolism in Patients With COVID-19. Ann. Intern. Med..

[56] Youd E., Moore L. (2020). COVID-19 autopsy in people who died in community settings: The first series. J. Clin. Pathol..

[57] Rapkiewicz A.V., Mai X., Carsons S.E., Pittaluga S., Kleiner D.E., Berger J.S., Thomas S., Adler N.M., Charytan D.M., Gasmi B. (2020). Megakaryocytes and platelet-fibrin thrombi characterize multi-organ thrombosis at autopsy in COVID-19: A case series. EClinicalMedicine.

[58] Martines R.B., Ritter J.M., Matkovic E., Gary J., Bollweg B.C., Bullock H., Goldsmith C.S., Silva-Flannery L., Seixas J.N., Reagan-Steiner S. (2020). Pathology and Pathogenesis of SARS-CoV-2 Associated with Fatal Coronavirus Disease, United States. Emerg. Infect. Dis..

[59] Barnes B.J., Adrover J.M., Baxter-Stoltzfus A., Borczuk A., Cools-Lartigue J., Crawford J.M., Daßler-Plenker J., Guerci P., Huynh C., Knight J.S. (2020). Targeting potential drivers of COVID-19: Neutrophil extracellular traps. J. Exp. Med..

[60] Li R., Yin K., Zhang K., Wang Y.Y., Wu Q.P., Tang S.B., Cheng J.D. (2020). Application Prospects of Virtual Autopsy in Forensic Pathological Investigations on COVID-19. Fa Yi Xue Za Zhi.

[61] Calabrese F., Pezzuto F., Fortarezza F., Hofman P., Kern I., Panizo A., von der Thüsen J., Timofeev S., Gorkiewicz G., Lunardi F. (2020). Pulmonary pathology and COVID-19: Lessons from autopsy. The experience of European Pulmonary Pathologists. Virchows Arch..

[62] Lindner D., Fitzek A., Bräuninger H., Aleshcheva G., Edler C., Meissner K., Scherschel K., Kirchhof P., Escher F., Schultheiss H.-P. (2020). Association of Cardiac Infection With SARS-CoV-2 in Confirmed COVID-19 Autopsy Cases. JAMA Cardiol..

[63] Bafunno V., Bury L., Tiscia G.L., Fierro T., Favuzzi G., Caliandro R., Sessa F., Grandone E., Margaglione M., Gresele P. (2014). A novel congenital dysprothrombinemia leading to defective prothrombin maturation. Thromb. Res..

[64] Santacroce R., Santoro R., Sessa F., Iannaccaro P., Sarno M., Longo V., Gallone A., Vecchione G., Muleo G., Margaglione M. (2008). Screening of mutations of hemophilia A in 40 Italian patients: A novel G-to-A mutation in intron 10 of the F8 gene as a putative cause of mild hemophilia a in southern Italy. Blood Coagul. Fibrinolysis.

[65] Sessa F., Bertozzi G., Cipolloni L., Baldari B., Cantatore S., D’Errico S., Di Mizio G., Asmundo A., Castorina S., Salerno M. (2020). Clinical-Forensic Autopsy Findings to Defeat COVID-19 Disease: A Literature Review. J. Clin. Med..

[66] Angileri F., Legare S., Marino Gammazza A., Conway de Macario E., JL Macario A., Cappello F. (2020). Molecular mimicry may explain multi-organ damage in COVID-19. Autoimmun. Rev..

[67] Huang S., Wang J., Liu F., Liu J., Cao G., Yang C., Liu W., Tu C., Zhu M., Xiong B. (2020). COVID-19 patients with hypertension have more severe disease: A multicenter retrospective observational study. Hypertens. Res..

[68] Ma L., Song K., Huang Y. (2020). Coronavirus Disease-2019 (COVID-19) and Cardiovascular Complications. J. Cardiothorac. Vasc. Anesth..

[69] Rubino F., Amiel S.A., Zimmet P., Alberti G., Bornstein S., Eckel R.H., Mingrone G., Boehm B., Cooper M.E., Chai Z. (2020). New-Onset Diabetes in Covid-19. N. Engl. J. Med..

[70] Henry B.M., Lippi G. (2020). Chronic kidney disease is associated with severe coronavirus disease 2019 (COVID-19) infection. Int. Urol. Nephrol..

[71] Middeldorp S., Coppens M., van Haaps T.F., Foppen M., Vlaar A.P., Müller M.C.A., Bouman C.C.S., Beenen L.F.M., Kootte R.S., Heijmans J. (2020). Incidence of venous thromboembolism in hospitalized patients with COVID-19. J. Thromb. Haemost..

[72] Zamboni P. (2020). COVID-19 as a Vascular Disease: Lesson Learned from Imaging and Blood Biomarkers. Diagnostics.

[73] Letko M., Marzi A., Munster V. (2020). Functional assessment of cell entry and receptor usage for SARS-CoV-2 and other lineage B betacoronaviruses. Nat. Microbiol..

[74] Shang J., Ye G., Shi K., Wan Y., Luo C., Aihara H., Geng Q., Auerbach A., Li F. (2020). Structural basis of receptor recognition by SARS-CoV-2. Nature.

[75] Messina G., Polito R., Monda V., Cipolloni L., Di Nunno N., Di Mizio G., Murabito P., Carotenuto M., Messina A., Pisanelli D. (2020). Functional Role of Dietary Intervention to Improve the Outcome of COVID-19: A Hypothesis of Work. Int. J. Mol. Sci..

[76] Giannopoulos G., Vrachatis D.A., Deftereos S.G. (2020). Myocardial Injury in COVID-19—Can We Successfully Target Inflammation?. JAMA Cardiol..

[77] Zuo Y., Yalavarthi S., Shi H., Gockman K., Zuo M., Madison J.A., Blair C., Weber A., Barnes B.J., Egeblad M. (2020). Neutrophil extracellular traps in COVID-19. JCI Insight.

[78] Tavazzi G., Pellegrini C., Maurelli M., Belliato M., Sciutti F., Bottazzi A., Sepe P.A., Resasco T., Camporotondo R., Bruno R. (2020). Myocardial localization of coronavirus in COVID-19 cardiogenic shock. Eur. J. Heart Fail..

[79] Monda V., Salerno M., Sessa F., Bernardini R., Valenzano A., Marsala G., Zammit C., Avola R., Carotenuto M., Messina G. (2018). Functional Changes of Orexinergic Reaction to Psychoactive Substances. Mol. Neurobiol..

[80] Pei G., Zhang Z., Peng J., Liu L., Zhang C., Yu C., Ma Z., Huang Y., Liu W., Yao Y. (2020). Renal involvement and early prognosis in patients with COVID-19 pneumonia. J. Am. Soc. Nephrol..

[81] Puelles V.G., Lütgehetmann M., Lindenmeyer M.T., Sperhake J.P., Wong M.N., Allweiss L., Chilla S., Heinemann A., Wanner N., Liu S. (2020). Multiorgan and Renal Tropism of SARS-CoV-2. N. Engl. J. Med..

[82] Wu Y., Xu X., Chen Z., Duan J., Hashimoto K., Yang L., Liu C., Yang C. (2020). Nervous system involvement after infection with COVID-19 and other coronaviruses. Brain. Behav. Immun..

[83] Solomon I.H., Normandin E., Bhattacharyya S., Mukerji S.S., Keller K., Ali A.S., Adams G., Hornick J.L., Padera R.F., Sabeti P. (2020). Neuropathological Features of Covid-19. N. Engl. J. Med..

[84] Li Y., Xiao S.Y. (2020). Hepatic involvement in COVID-19 patients: Pathology, pathogenesis and clinical implications [Review]. J. Med. Virol..

[85] Ellinghaus D., Degenhardt F., Bujanda L., Buti M., Albillos A., Invernizzi P., Fernández J., Prati D., Baselli G., Asselta R. (2020). Genomewide Association Study of Severe Covid-19 with Respiratory Failure. N. Engl. J. Med..

[86] Marino Gammazza A., Légaré S., Lo Bosco G., Fucarino A., Angileri F., Conway de Macario E., Macario A.J., Cappello F. (2020). Human molecular chaperones share with SARS-CoV-2 antigenic epitopes potentially capable of eliciting autoimmunity against endothelial cells: Possible role of molecular mimicry in COVID-19. Cell Stress Chaperones.

